# (−)-Epigallocatechin Gallate Stability in Ready-To-Drink (RTD) Green Tea Infusions in TiO_2_ and Oleic-Acid-Modified TiO_2_ Polylactic Acid Film Packaging Stored under Fluorescent Light during Refrigerated Storage at 4 °C

**DOI:** 10.3390/foods10040723

**Published:** 2021-03-29

**Authors:** Naerin Baek, Young Kim, Susan Duncan, Kristen Leitch, Sean O’Keefe

**Affiliations:** 1 Department of Food Science and Technology, Virginia Tech, Blacksburg, VA 24061, USA; nbaek@vt.edu (N.B.); duncans@vt.edu (S.D.); kaleitch@vt.edu (K.L.); 2 Department of Sustainable Biomaterials, Virginia Tech, Blacksburg, VA 24061, USA; ytkim@vt.edu

**Keywords:** titanium dioxide (TiO_2_), polylactic acid (PLA), green tea infusion, (−)-epigallocatechin gallate (EGCG), light protection, fluorescent light

## Abstract

The light-protective effectiveness of titanium dioxide polylactic acid (TiO_2_ PLA) nanocomposite films (T-PLA) and oleic-acid-modified (OA_TiO_2_PLA) nanocomposite films was investigated in ready-to-drink (RTD) green tea infusions in oxygen-impermeable glass packaging. The stability of (−)-epigallocatechin gallate (EGCG) was evaluated in RTD green tea infusions in glass packaging covered with PLA (polylactic acid), T-PLA and OT-PLA under fluorescent light during 20 days of storage at 4 °C. Levels of EGCG and color change of RTD green tea infusions were determined. In addition, sensory tests for difference were conducted on green tea infusions in glass packaging without and with complete light protection during 10 days of storage at 4 °C. Of the panelists, 60% noticed sensory differences in the RTD green tea infusion in two different packaging conditions during 10 days of storage under fluorescent light by a triangle test (*p* < 0.05). During 20 days of storage, levels of EGCG with complete light protection decreased by 10.8% (0.73 mg/mL), and there was a 42.2% loss of EGCG (0.48 mg/mL) in RTD green tea infusions in the glass packaging covered by PLA film. Finally, 3% T-PLA preserved higher levels of EGCG in RTD green tea infusions compared to 1% T-PLA and OT-PLA.

## 1. Introduction

There has been a growing consumption of green tea (*Cameilla sinesis*) due to potential health benefits associated with tea catechins. They may reduce the risk of cardiovascular diseases, strokes and cancers [1,2]. Green tea is a nonfermented tea containing higher amounts of tea catechins than any other major types of tea such as oolong tea (semifermented tea) or black tea (fermented) tea [3]. Around 22% of world tea production is green tea, and China is the main producer. Green tea contains polyphenol compounds such as (+)-catechin (C), (−)-epicatechin (EC), (−)-epicatechin gallate (ECG), (−)-epigallocatechin (ECG), (−)-epigallocatechin gallate (EGCG), (−)-gallocatechin gallate (GCG) and (−)-gallocatechin (GC). (−)-Epigallocatechin gallate (EGCG) is not only the most abundant catechin but also a potent antioxidant and a green tea quality indicator [4].

Ready-to-drink (RTD) green tea infusions are widely available in various packaging materials such as glass, plastic and aluminum containers, yet EGCG, which is a major bioactive compound, is found in lower amounts in commercial RTD than in prepared green tea using traditional brewing methods [5,6]. EGCG is easily susceptible to epimerization and oxidation by light, oxygen, brewing methods, storage conditions and packaging materials [6,7,8,9].

Kim, Welt and Talcott (2011) reported the stability of tea catechin was influenced significantly by oxygen contact [5]. Green tea infusions in glass bottles, which have the lowest oxygen permeation rates, retained the most tea catechins compared to green tea stored in polyethylene terephthalate (PET) bottles and retortable pouches [5]. In addition, the degradation of EGCG and EGC influences the sensory quality of green tea infusions. EGCG and other tea catechins in green tea infusion drinks should be maintained at the highest levels possible before consumption to provide healthful bioactive compounds and a delicate green tea sensory quality [4]. Green tea flavor depends on the growing conditions of the tea as well as the processing methods, which can be pan fried or steam heated. A green tea flavor lexicon includes green (asparagus, beany, celery, green herb-like), brown (brown spice, nutty tobacco) and fruity/floral (fruity, floral, citrus) characteristics (Lee and Chambers) [10].

Fresh tea leaves contain mainly photosensitized pigments such as chlorophylls (0.2–0.6% dry weight) and carotenoids [11]. Chlorophylls absorb light at wavelengths of 430 and 660 nm [12]. Light has a detrimental effect on green tea flavor; when green tea was exposed under 2000 lux light for one month, the green tea infusion had unacceptable off-flavors [11].

The stability of EGCG and other tea catechins in green tea infusions has been extensively studied. However, studies related to light influence on the photochemical stability of EGCG in green tea infusions have been limited [6,8]. Investigating the photochemical behavior of EGCG levels in green tea infusions by light exposure is important to prevent the decomposition of EGCG and maintain the major antioxidant as well as delicate green tea flavor in suitable packaging during retail display.

TiO_2_ is well known as a low-cost, nanostructured filler for polymer nanocomposites in food packaging applications due to enhancing barrier properties as well as providing color, UV-light protection and antibacterial effects under UV-light illumination [12,13,14]. TiO_2_ has been added to biodegradable polylactic acid (PLA), which is classified as generally regarded as safe (GRAS), for direct-contact materials in food packaging application by the U.S. Food and Drug Administration [15].

PLA films containing TiO_2_ were created to examine TiO_2_’s light-protective effectiveness for EGCG in a green tea model under fluorescent light illumination. Due to the low miscibility of TiO_2_ with PLA solutions, TiO_2_ was modified with oleic acid to improve its miscibility with PLA solution. In this study, TiO_2_ PLA and OA_TiO_2_ PLA nanocomposite films were prepared [16] to investigate packaging impacts on the stability of EGCG in an RTD green tea infusion model under fluorescent light. In an initial study, we investigated sensory differences and EGCG levels in RTD green tea infusions stored in clear glass and light-blocked packaging exposed to fluorescent light during 10 days of storage at 4 °C. In a second study, levels of EGCG in RTD green tea in glass bottles were overwrapped by PLA, foil (complete light protection) and 3% T-PLA and 1% and 3% OT-PLA during 20 days of storage at 4 °C under fluorescent light.

## 2. Materials and Methods

### 2.1. Materials

Dragon Well green tea was purchased from an online tea merchant (enjoyingtea.com). Spring water (6 L) and granulated white sugar were obtained from a local supermarket (Kroger, Blacksburg, VA, USA). Food-grade anhydrous citric acid was received from ADM (Decatur, IL). (−)-Epigallocatechin gallate (EGCG) was obtained from Sigma-Aldrich Chemical Co. (St. Louis, MO, USA). Methanol, phosphoric acid and acetonitrile (HPLC grade) were purchased from Fisher Scientific (Pittsburgh, PA, USA). Food-grade Star San^TM^ was used as a sanitizer for glass bottles used for green tea drink samples.

Study 1. Light effects on sensory differences and degradation of (−)-epigallocatechin gallate (EGCG) in ready-to-drink green tea infusions during refrigerated storage at 4 °C.

### 2.2. Ready-To-Drink (RTD) Green Tea Infusion Preparation

Fourteen clean glass bottles (340 mL each) were washed, air-dried then sanitized. Dragon Well green tea (60 g) was brewed with 6 L of spring water at 90 °C for 5 min, then sugar (216 g) was added. Citric acid (6 g) was added to the green tea infusion to create a pH of less than 4. The RTD green tea infusion was filled hot into sanitized glass bottles, and then the bottles were placed upside down for 2 min (hot fill). The green tea was then cooled in an ice-water bath.

### 2.3. Storage Conditions—Experiment 1

RTD green tea infusions were stored under different packaging conditions: light-blocked and light-exposed glass. Control light-blocked samples of RTD green tea infusions were contained in light-blocked glass bottles (7 bottles) covered by aluminum foil. Treatment light-exposed samples of RTD green tea samples did not have the aluminum foil wrap (7 bottles). Approximately 2000–2500 lux of fluorescent light produced by Sylvania bulbs (designer cool white 30 W, F30T12/DCW/RS, Mississuaga, ON, Canada) was exposed to fourteen glasses bottles containing RTD green tea infusion in a walk-in cooler at 4 °C during 10 days of storage. The positions of the glass bottles were rotated every 24 h to ensure even light exposure. All glass bottles were removed from fluorescent light after 10 days of storage at 4 °C 3 h before conducting a sensory triangle test and HPLC analysis. RTD green tea infusion in the light-blocked glass overwrapped foil was transferred into a 3 L plastic bottle for blending. The other RTD green tea infusion in seven bottles without the aluminum foil was transferred into a 3 L plastic water bottle to blend as well. One ounce of RTD green tea infusion was filled in two-ounce cups codded with randomly assigned 3-digit numbers 2 h prior to the sensory triangle test, capped and stored at 4 °C of refrigeration in a sensory preparation lab until the samples were served to panelists.

### 2.4. High-Performance Liquid Chromatography (HPLC) Analysis

ECGC level was determined using an Agilent Technologies model 1200 series HPLC system (Santa Clara, CA, USA) following the procedure described by Yoshida and others (1999) [17]. A C-18 Luna 5 μm (25 cm × 4.9 mm i.d.) column fitted with a guard column (Phenomenex, Torrence, CA, USA) was used for the analysis. The mobile phase consisted of solvent A (95.45% distilled water, 4.5% acetonitrile and 0.05% O-phosphoric acid) and phase B (49.95% distilled water, 50% acetonitrile and 0.05% O-phosphoric acid). The flow rate was set to 1.0 mL/min, and the column temperature was maintained at 40 °C.

### 2.5. Sensory Analysis

A sensory triangle test for differences was conducted in the sensory lab of the Human and Agriculture Biosciences Building at Virginia Tech. Panelists (*n* = 58) ranged in age from 18 to 24 (59%), 25 to 34 (31%), 35 to 45 (7%) and 45+ (3%). Of the panelists, 57% were women, and 83% of the panelists had positive responses for liking the consumption of RTD green tea. Of the panelists, 10% consumed green tea at least one time a day, and 57% of the panelists consumed green tea at least once a month. Training was not required, but the panelists were familiar with the triangle test method. Panelists were recruited from undergraduates and graduate students, faculty and staff from the Departments of Food Science and Technology and Biological System Engineering at Virginia Tech. Panelists were seated in individual booths and evaluated samples under red light in order to mask the colors of RTD green tea infusion samples and eliminate bias [18]. The use of colored lighting was because there was an obvious change in green color in samples exposed to light, but we were interested in knowing if there were taste/aroma differences among the samples. IRB approval (IRB No. 14-278) was received from the Virginia Tech Office of Research Compliance. The panelists filled out human subject consent forms (IRB No. 14-278) before proceeding with the evaluation. One set of three samples (*n* = 3) was presented simultaneously in a balanced order of presentation using a complete block design. The temperature of the RTD green tea infusion samples was controlled at 4 °C to minimize the flavor change of samples by temperature as the samples were stored in the refrigerator just before serving the samples to panelists. One set of three samples compared light-blocked RTD green infusion treatment (A) to light-exposed RTD green tea infusions (B). The samples were presented in a random order to panelists using different orders to minimize bias [18]. The panelists were asked to taste three samples from left to right and choose a different tasting sample within three samples and asked to indicate “odd taste sample” on the scorecards [18]. After completing one set of triangle tests, panelists were requested to fill out a survey including demographics, age, race, frequent consumption and self-reported preference of RTD green tea infusion (see Appendix A).

### 2.6. Experimental Design and Statistical Analysis

For the triangle sensory difference test, statistical parameters were defined at α = 0.05, β = 0.1 and P_d_ (proportion of discriminators) = 30%. The minimum number of subjects needed was 53 and we had 58.

Analysis of variance was used to determine if the EGCG level in RTD green tea infusions stored in two different packaging conditions was significantly different after 10 days of storage under light exposure. The significant difference in mean values (*n* = 2) of the two treatments was compared by a Student’s *t*-test. The null hypothesis was rejected, and the mean values were significantly different from each other when *p* > 0.05.

Study 2. (−)-Epigallocatechin gallate stability in ready-to-drink green tea infusions in TiO_2_ and OA_TiO_2_ PLA film packaging models under fluorescent lighting during refrigerated storage at 4 °C.

### 2.7. Ready-To-Drink (RTD) Green Tea Infusion Preparation

Dragon well green tea (8.42 g) was brewed with 850 mL of spring water at 90 °C for 5 min, then 28.8 g of sucrose and 0.81 g of citric acid were added. The pH of the tea preparation was 3.74. The oxygen-impermeable glass bottles were used to investigate only the light-blocking effects of the films for the green tea infusion. A 15 mL volume of hot (82–85 °C) RTD green tea infusion was filled into 20 mL glass bottles and capped tightly, then the bottles were placed upside down for 2 min. The bottles containing the RTD green tea were then cooled in an ice-water bath. Films for testing (foil, PLA, 1% T-PLA, 1% OT-PLA, 3% T-PLA and 3% OT-PLA) were cut into uniform sizes of 5 cm × 9 cm for covering the glass bottles. The thickness of the three sections of each film was measured by a digital micrometer (Mitutoyo 700-118-20 Digital Thickness Gage, Kawasaki, Japan), and then we averaged the thickness of the films (Table 1). The glass bottles were covered by one layer of aluminum foil (complete light blocking), PLA (light exposed), 1% T-PLA, 1% OT-PLA, 3% T-PLA and 3% OT-PLA. The TiO_2_ levels in PLA and oleic-modified TiO_2_ treatments used were described earlier [16].

### 2.8. Storage Conditions

Fluorescent light bulbs (1990 lux ± 365) produced by Sylvania (designer cool white 30 W, F30T12/DCW/RS, Mississuaga, ON, Canada) provided light exposure to the glass bottles containing RTD green tea infusion in a walk-in cooler at 4 °C during 20 days. The light intensities and light spectrum were measured by using a Upertek MK350S (1B) (Miaoli County, Taiwan). The positions of the glass bottles were randomly rotated every 24 h. Eighteen (6 treatments in triplicate) bottles containing RTD green tea infusions were taken from the storage every 10 days for EGCG analysis. The colors of the green tea infusion were measured on Days 0 and 20.

### 2.9. HPLC Analysis

ECGC level was determined using an Agilent Technologies model 1260 series HPLC system (Santa Clara, CA, USA) followed by the procedure described by Yoshida and others (1999) [18]. A Nucleosil 100-5 C-18 (25 cm × 4.6 mm i.d.) column, obtained from Machery-Nagel (Düren, Germany), was used for the analysis. The solvent consisted of mobile phase A (95.45% distilled water, 4.5% acetonitrile and 0.05% O-phosphoric acid) and mobile phase B (49.95% distilled water, 50% acetonitrile and 0.05% O-phosphoric acid). The flow rate was set to 1.0 mL/min, and the column temperature was maintained at 40 °C.

### 2.10. Color Analysis

A colorimeter (Minolta Chroma Meter CR-200, Osaka, Japan) was used to analyze the color changes of RTD green tea infusions on Days 0 and 20. Calibration using a white calibration plate (CR-A44) was conducted first. The color values of the RTD green tea infusions in the glass packaging covered by the films were reported as L* = lightness (0 = black, 100 = white), a* (+a* = redness and −a* = greenness) and b* (+b* = yellowness and −b* = blueness).

### 2.11. Statistical Analysis

One-way Analysis of Variance was used to determine if the EGCG concentration changed in RTD green tea infusion stored under six different packaging models under the fluorescent light treatments. Significant differences between mean values were determined by using Tukey’s test at *p* < 0.05.

## 3. Results and Discussion

Study 1. Light effects on sensory differences and the degradation of (−)-epigallocatechin gallate (EGCG) in ready-to-drink green tea infusion.

The triangle test was conducted to determine if panelists could detect aroma or taste differences between RTD green tea in clear glass packaging and in light-blocked packaging when exposed to light. Since we used red light to mask color differences, color was not evaluated as a difference criterion by the panelists. In order to reduce Type 1 error, the alpha value was chosen as 0.05 [18]. The null hypothesis was rejected since the number of correct responses for the triangle test was 35 out of 58 respondents at α = 0.05, β = 0.1 and P_d_ = 30%. Of the panelists, 60% were able to correctly identify the odd sample between RTD green tea samples stored in the packaging with and without light blocking during storage. From the results of the triangle discrimination sensory test, there was a significant difference in RTD green tea infusions stored in two different packaging conditions when exposed to light during storage. Since we used red light to mask color differences, the panelists used other differences in the samples to identify the odd sample.

Good-quality green tea has a well-balanced flavor with bitterness, astringency and a sweet aftertaste [11,19]. Narukawa, Kimata, Noga and Wantanbe (2010) reported that tea catechins caused astringency and bitterness in tea; in particular, EGC and EGCG were recognized as having bitter and astringency sensory attributes in green tea [20]. However, the bitterness and astringency of green tea (determined by sensory perception) decreased after green tea extracts were stored at 50 °C during accelerated storage because of degradation of the phenolic contents [21]. The degradation of EGCG in RTD green tea infusions during the storage by HPLC analysis supported the sensory triangle test result.

HPLC analysis

Light effects on the degradation of EGCG in RTD green tea infusions were observed. HPLC analysis was conducted to evaluate the stability of EGCG in RTD green tea infusions retained in clear glass (light-exposed packaging) and aluminum-foil-wrapped glasses (light-blocked packaging) exposed to fluorescent light (2000–2500 lux) during 10 days storage at 4 °C. A peak of EGCG on the HPLC chromatogram was identified and quantified by a comparison of retention times and areas of the peak using the authentic standard of EGCG. The concentration of EGCG in RTD green tea infusions was attained by an external standard curve created by an authentic EGCG standard solution (mg/mL) diluted five times with distilled water using serial dilution.

Initial concentrations of EGCG in RTD green tea infusions (0.51 mg/mL) before storage were decreased by 9% (0.47 mg/mL) and 16% (0.43 mg/mL) in glass packaging with and without light blocking, respectively, at 4 °C for 10 days of storage under light exposure (Table 2). The null hypothesis (μEGCG in light-blocked glass = μEGCG levels in clear glass) was rejected by one-way Analysis of Variance (ANOVA) (*p* < 0.05), and mean values of the levels of EGCG between the two different packaging conditions were compared using a Student’s *t*-test. Significant differences in levels of EGCG were found between the two different packaging conditions at 4 °C during 10 days of storage (*p* < 0.05).

Study 2. (−)-Epigallocatechin gallate stability in ready-to-drink green tea infusions in TiO_2_ and OA_TiO_2_ PLA film packaging models under fluorescent lighting during refrigerated storage at 4 °C.

HPLC analysis

EGCG stability in RTD green tea infusions retained in glass bottles covered by foil, PLA, 1% T-PLA, 3% T-PLA, 1% OT-PLA and 3% OT-PLA under fluorescent light (1990 lux ± 365) was evaluated during 20 days of storage at 4 °C. EGCG was identified and quantified according to the HPLC analysis described in Study 1.

**Figure 1:**
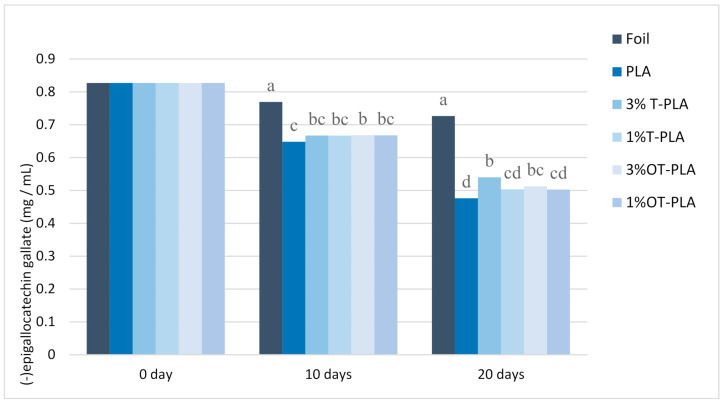
(−)-Epigallocatechin gallate concentrations in green tea infusions in glass bottles covered by foil, PLA, 3% T-PLA, 1% T-PLA, 3% OT-PLA and 1% OT-PLA nanocomposite films at Days 0, 10 and 20 under fluorescent light illumination at 4 °C. Significant differences between the samples are expressed with different superscript letters above the data by Tukey’s analysis with *p* < 0.05. Bars represent the means of three replicates, and significant differences (*p* < 0.05) are presented by different letters.

Before storage under fluorescent light, the initial concentration of EGCG in the RTD green tea infusion was 0.83 mg/mL. After 10 days of storage, amounts of EGCG were varied in RTD green tea depending on the packaging. EGCG in RTD green tea infusions was the most stable in the glass bottle covered by aluminum foil (complete light-protective film). The amount of EGCG in RTD green tea in the packaging covered by PLA (transparent light-exposed film) shows the largest decrease of EGCG by 21.7% (0.65 mg/mL). The light-protected packaging with a foil wrap retained the most amount of EGCG in RTD green tea (0.77 mg/mL) on Day 10. On Day 20, the ECGC level in RTD green tea infusions in the glass packaging covered by PLA was decreased by 42.2% (0.48 mg/mL) from an initial ECGC level in RTD green tea infusions. The results showed that the levels of EGCG in RTD green tea infusions in the packaging covered by 3% T-PLA and 3% OT-PLA were retained at higher amounts than 1% T-PLA and 1% OT-PLA. Precisely 3% of the T-PLA packaging preserved higher levels of EGCG in RTD green tea infusions than 3% OT-PLA and 1% T-PLA, and 1% OT-PLA had slight light protection for EGCG in RTD green tea infusions during 20 days of storage, as seen in Figure 1. The TiO_2_ treatments were selected because this common plastic additive helps to block light [16], which we hypothesized would improve stability by preventing photooxidation, perhaps catalyzed by the photosensitizer, chlorophyll. The oleic acid modification improved the compatibility of TiO_2_ with the PLA polymer but also impacted clarity somewhat [16], which negatively affects light blocking.

**Figure 2:**
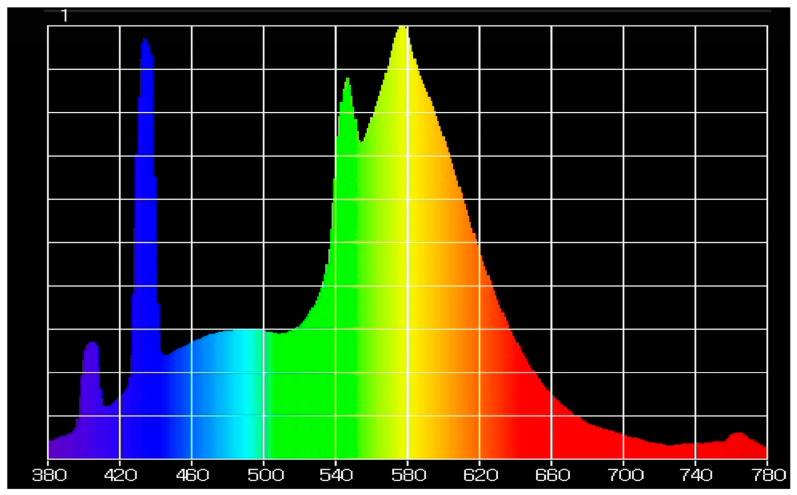
The light spectrum from the fluorescent bulbs was measured at 2393 lux by using a Upertek MK350S.

Oxygen and light influence the degradation of tea catechins and produce off-flavors of green tea [4,5]. Scalia, Marchetti and Bianchi (2013) reported that EGCG in a hydrophilic cream model system was degraded by 76.9 ± 3.7% under 1 h solar simulator emission at 500 W/m^2^ with controlled temperature below 37 °C [8,22]. However, the addition of vitamin C in the hydrophilic cream reduced the loss of the EGCG level by 20.4 ± 2.7% compared to the cream without vitamin C [22]. The fluorescent light used in our experiment had a high light power at 430, 550 and 580 nm in the light spectrum (Figure 2).

Chlorophyll has light absorption peaks at wavelengths of 430 and 660 nm [12]. As a result, chlorophyll might have acted as a photosensitizer in our model system. If this happened, chlorophyll photooxidation might have degraded catechins in the green tea. The 3% T-PLA films had much higher light absorption in the visible region than OT-PLA films. EGCG levels in RTD green tea infusions in the glass packaging covered by 3% T-PLA were higher than the T-PLA and OT-PLA packaging. Further studies are needed to understand the specific wavelengths of light that cause the degradation of catechins in tea.

Because RTD green tea drinks usually have a long retail shelf life in retail stores and are displayed in retail cases under light, the use of suitable packaging is important to preserve EGCG and other tea catechins and keep delicate green tea flavors. Consumers prefer to see foods through the packaging when they make food selections. The transparency of OT-PLA was quite similar to PLA due to the well-dispersed TiO_2_ nanoparticles that result from the surface modification in PLA. However, OT-PLA had less effective protection of EGCG compared to T-PLA when the same concentrations of TiO_2_ or oleic-acid-modified TiO_2_ (OA_TiO_2_) were incorporated into PLA. This study suggests that higher amounts of TiO_2_ loading in PLA retained levels of EGCG better because of better light protection. However, protection was less than the protection of EGCG by foil.

Color changes

Color changes of the green tea infusion between six different packaging conditions were measured on Days 0 and 20 (Table 3).

On Day 20, the RTD green tea infusion in the packaging with complete light protection had the lowest a-value, which means that the color of the RTD green tea was the greenest and least red among the RTD green tea in six different packaging conditions, whereas RTD green tea in the PLA packaging had the highest a-value, which means it was the least green and the most red. During 20 days of storage, the color of the green tea was changed significantly. These results showed that higher EGCG degradation in RTD green tea infusions in PLA packaging and 1% T-PLA and 1% OT-PLA had more reddish color than less degradation of EGCG in RTD green tea infusions in the packaging such as foil and 3% T-PLA. The color change of the green tea was dependent on the films and storage time under light exposure. Wang, Kim and Lee (2000) observed that the oxidation of tea phenolic compounds led to the development of brown color in green tea extract during 12 days of storage at 50 °C during accelerated storage conditions [21]. The a-value of the color change results agreed with previous research findings that green tea color becomes more reddish as a result of the oxidation of polyphenolic compounds. Chlorophyll is a green pigment in green tea, and its amount decides the final color of green tea infusion [23]. Light may degrade chlorophyll in RTD green tea infusions during storage because light absorptions of the photosensitizer chlorophyll are at the wavelength of 430 and 660 nm [12,23]. Hence, the light may reduce the green color of RTD green tea infusions in the packaging during storage.

## 4. Conclusions

EGCG levels in RTD green tea infusions in light-exposed glass packaging significantly decreased during 10 days of storage at 4 °C under fluorescent light. The taste/aroma of the green tea in light-blocking and light-exposed packaging during 10 days of storage was also significantly different. Higher levels of TiO_2_ incorporated into PLA more effectively and retarded EGCG degradation. OT-PLA was less effective than T-PLA for light protection assessed by EGCG stability in RTD green tea infusions. The color of RTD green tea infusions in glass packaging covered by PLA displayed less green than RTD green tea infusions in glass packaging with complete light protection. Preservation of EGCG and other tea catechins is essential to retain the antioxidant capacity and delicate green tea taste, and commercial RTD green tea infusions should be sold in appropriate packaging that provides not only a high oxygen barrier but also light protection.

## Figures and Tables

**Table 1:** Thickness (mean ± standard deviation) of Polylactic Acid (PLA), 3% TiO_2_ PLA nanocomposite films (T-PLA), 1% T-PLA, 3% oleic-acid-modified OA_TiO_2_PLA nanocomposite films (OT-PLA) and 1% OT-PLA nanocomposite films covered the glass bottles (*n* = 6).

Sample (*n* = 6)	Mean (μm) ± Std. Dev.
PLA	70.6 ± 15.1
3% T-PLA	62.2 ± 8.06
1% T-PLA	61.1 ± 9.61
3% OT-PLA	66.7 ± 4.7
1% OT-PLA	62.2 ± 10.3

**Table 2:** Changes of EGCG (mg/mL) in ready-to-drink green tea infusions stored in light-blocking (LB) glass packaging and clear glass light-exposed (EX) packaging under light exposure at 4 °C during 10 days of storage. The concentration (mg/mL) of LB and EX is expressed as means (*n* = 2). A significant difference between the two treatments is expressed with different superscript letters (a or b) next to the data (*p* < 0.05).

Storage Time	EGCG (mg/mL)	
	LB	EX
0 days	0.51	0.51
10 days	0.47 ^a^	0.43 ^b^

**Table 3:** Color changes (mean ± standard deviation) in L*value (L*: 0 = black, 100 = white), a- and b-values of ready-to-drink (RTD) green tea in the glass bottles covered by foil, PLA, 3% T-PLA, 1% T-PLA, 3% OT-PLA and 1% OT-PLA nanocomposite films during storage of 0 and 20 days at 4 °C under fluorescent light. Different letters next to the data indicate statistical differences between L, a- and b-values of the color at *p* < 0.05.

			L-values			
Sample	Foil	PLA	3% T-PLA	1% T-PLA	3% OT-PLA	1% OT-PLA
0 days	92 ± 0.8	92 ± 0.8	92 ± 0.8	92 ± 0.8	92 ± 0.8	92 ± 0.8
20 days	97 ± 1 ^a^	97 ± 0.1 ^a^	95 ± 0.1 ^bc^	96 ± 0.1 ^ab^	96 ± 0.1 ^ab^	94 ± 0.4 ^c^
			a-values			
Sample	Foil	PLA	3% T-PLA	1% T-PLA	3% OT-PLA	1% OT-PLA
0 days	0.36 ± 1	0.36 ± 1	0.36 ± 1	0.36 ± 1	0.36 ± 1	0.36 ± 1
20 days	−1.38 ± 0.1 ^c^	−0.46 ± 0.4 ^a^	−1.14 ± 0.4 ^bc^	−1.03 ± 0.2 ^bc^	−0.72 ± 0.2 ^ab^	−0.69 ± 0.3 ^ab^
			b-values			
Sample	Foil	PLA	3% T-PLA	1% T-PLA	3% OT-PLA	1% OT-PLA
0 days	3.8 ± 0.2	3.8 ± 0.2	3.8 ± 0.2	3.8 ± 0.2	3.8 ± 0.2	3.8 ± 0.2
20 days	2.4 ± 0.1 ^a^	−2.0 ± 0.1 ^c^	−1.0 ± 0.2 ^b^	−1.3 ± 0.2 ^bc^	−1.9 ± 0.1 ^c^	−1.3 ± 0.3 ^bc^

## Data Availability

Raw data can be obtained by contacting the PI O’Keefe.

## Appendix A

**APPENDICES**
**Questionnaire for Sensory Study**
**Please answer all of the following questions.**
**Gender**
Male
Female
**Age**
18–24
25–34
35–45
45 and older
**Ethnicity origin (or Race): Please specify your ethnicity.**
Hispanic
Caucasian
African or African American
Native American
Asian
Other
**Do you like to drink a green tea?**
Yes
No
**How often do you consume green tea drinks?**
More than one time in a day
At least one time in a day
Three times in a week
Once in two weeks
Once in a month
Seldom
Never
**Scorecards**
Panelist Number___________
Sample: Ready to Drink Green Tea Infusion
Characteristic Studied: Flavor
**Instructions:**
Taste samples from left to right. Two samples are identical; determine which one is the odd sample. If no difference is apparent, enter your best guess.
**Sample Codes** 653, 298, 455
Please circle or check mark on the odd sample.
Thank you for completing this sensory test. Please return the tray and scorecard to the researchers by sliding it through the booth. Please wait for demographics survey.
**Virginia Polytechnic Institute and State University**
**Informed Consent for Participants in Research Projects Involving Human Subjects (Sensory Evaluation)**
**Title Project:** Sensory Differences in Ready to Drink Green Tea Infusion during Storage at 4 °C
**Investigators**: Susan E. Duncan, PhD, Sean O’Keefe, PhD, Naerin Baek, and Kristen Leitch
**I. Purpose of this Research/Project**
You are invited to participate in a study to determine whether consumers can detect overall sensory difference of green tea affected by packaging during storage at 4 °C.
**II. Procedures**
You will be evaluating one set of green tea drinks for sensory quality. The set will have three samples. You will identify a sample that is different based on flavor, within one set and respond using a scorecard or a touch screen. You are encouraged to participate in the session, if possible.
**III. Risks**
There are only minimal risks associated to study participants with this project. Individuals with green tea allergy, caffiene and acid sensitivity, iron deficiency anemia or pregnancy risk may have risks.
**IV. Benefits**
The goal of this research is to determine if packaging and storage condition affect sensory difference of green tea drinks during storage at 4 °C. The information from this study will help determine appropriate packaging and storage condition for green tea drinks.
**V. Extent of Anonymity and Confidentiality**
The results of your performance as a panelist will be kept strictly confidential except to the investigator. Individual panelists will be referred to by a code number for data analyses and for any publication of the results.
**VI. Compensation**
You will be compensated with a small edible treat and a canned food at the end of the session.
**VII. Freedom to Withdraw**
If you agree to participate in this study, you are free to withdraw from the study at any time without penalty. Please withdraw from the study if you are less than 18 years of age.
**VIII. Subject’s Responsibilities**
I voluntarily agree to participate in this study. I have the following responsibilities:
● Evaluate 1 set (3 samples of green tea drinks, as presented, and provide answers using a score card or touch) screen.
**IX. Subject’s Permission**
I have read the consent form and conditions of this project. I have had all my questions answered. I hereby acknowledge the above and give my voluntary consent:
Date: _____________________
Subject Signature: _____________________________________________
Subject Printed Name: _________________________________________
- - - - - - - - - - - For Human Subject to Keep - - - - - - - - - - - -
Should I have any pertinent questions about this research or its conduct, and research subjects’ rights, and whom to contact in the event of a research-related injury to the subject. I may contact:
Susan Duncan, Faculty/Investigator (540) 231-8675;
duncans@vt.edu
Sean O’Keefe, Faculty/Investigator (540) 231-4437;
okeefes@vt.edu
Kristen Leitch, Graduate Student/Investigator (540) 231-8675;
kaleitch@vt.edu
Naerin Baek, Graduate Student/Investigator (540) 231-8675;
nbaek@vt.edu
David Moore
Chair, Virginia Tech Institutional Review (540) 231-4991;
Board for the Protection of Human Subjects moored@vt.edu
Office of Research Compliance
Blacksburg, VA 24061
